# Supplementary material for: Influences of Vitamin B12 Supplementation on Cognition and Homocysteine in Patients with Vitamin B12 Deficiency and Cognitive Impairment
Source: Nutrients. 2022 Apr 2;14(7):1494. doi: 10.3390/nu14071494 (PMC9002374; doi:10.3390/nu14071494)
Supplement: Supplementary file 1 [file nutrients-14-01494-s001.zip › nutrients-1609925-supplementary.pdf]

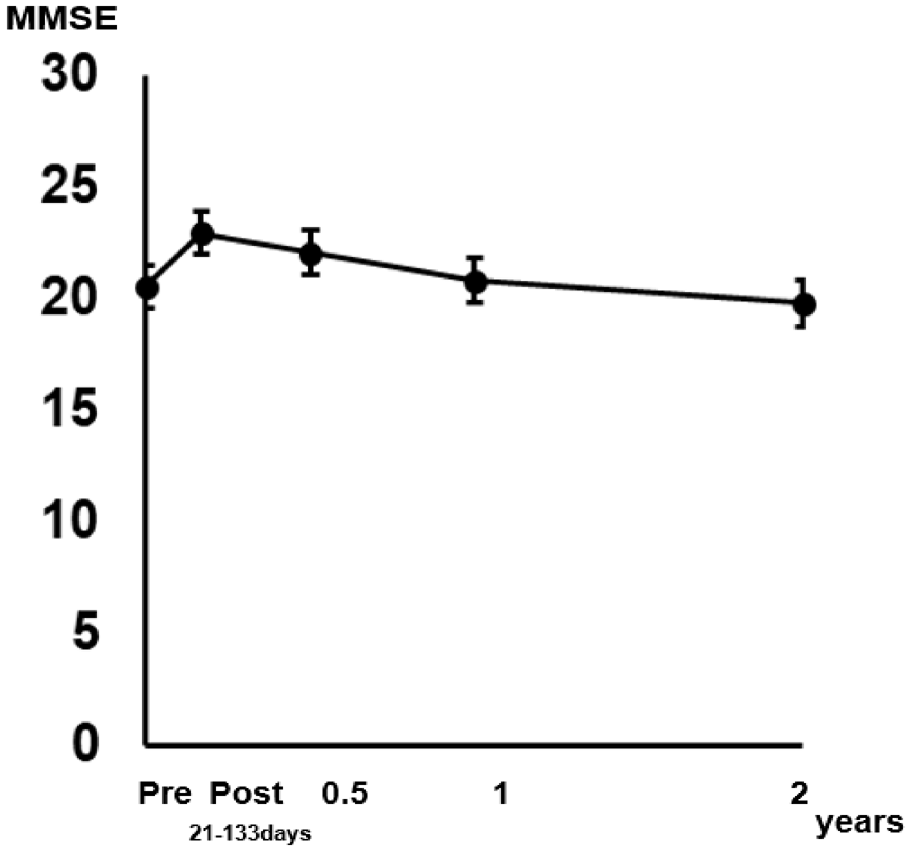

Supplementary Figure S1. Long duration of Mini-Mental State Examination (MMSE) score follow-up at 0.5 year, 1 year, and 2 year later was analyzed by the Generalized mixed model (GLMM).

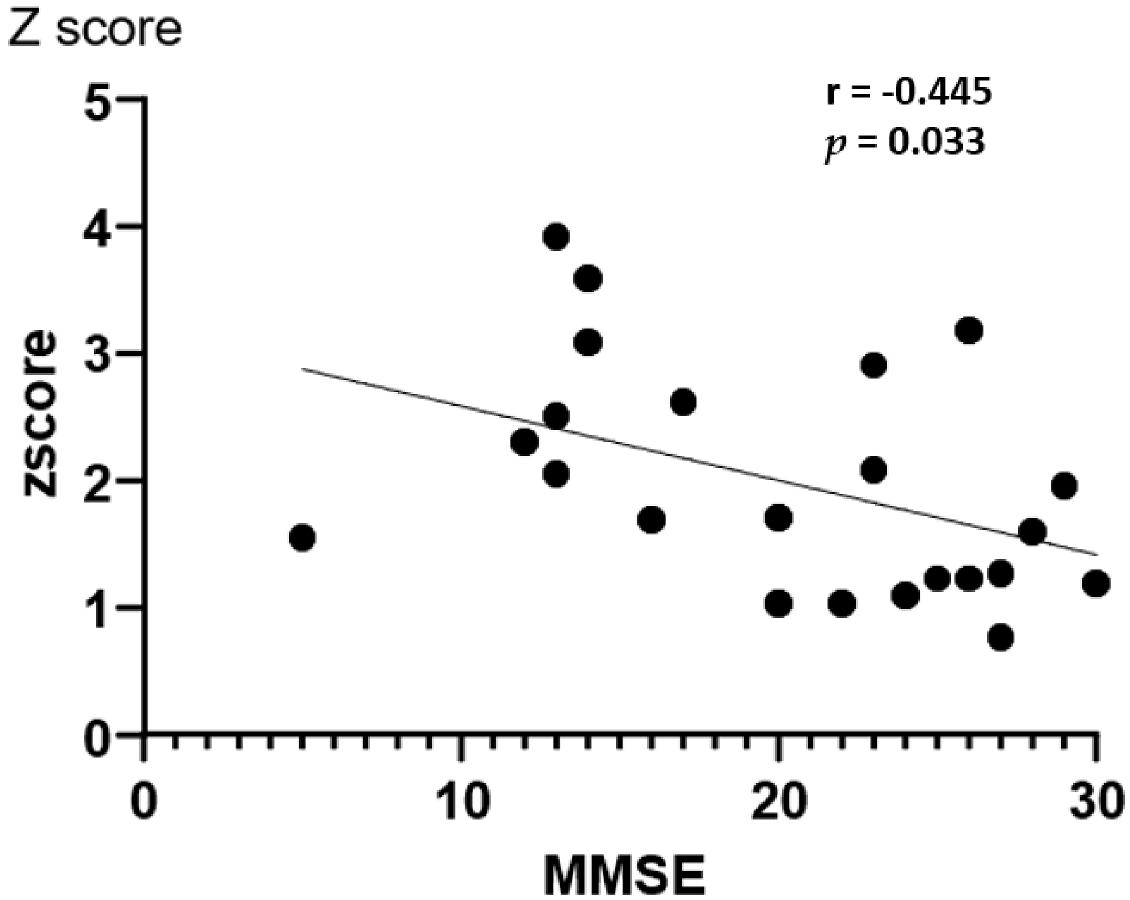

Supplementary Figure S2. Inverse correlation is shown between mini-mental state examination (MMSE) score and hippocampal atrophy estimated by VSRAD z-score (r = − 0.445, p = 0.033) was analyzed by the Pearson's correlation coefficient.

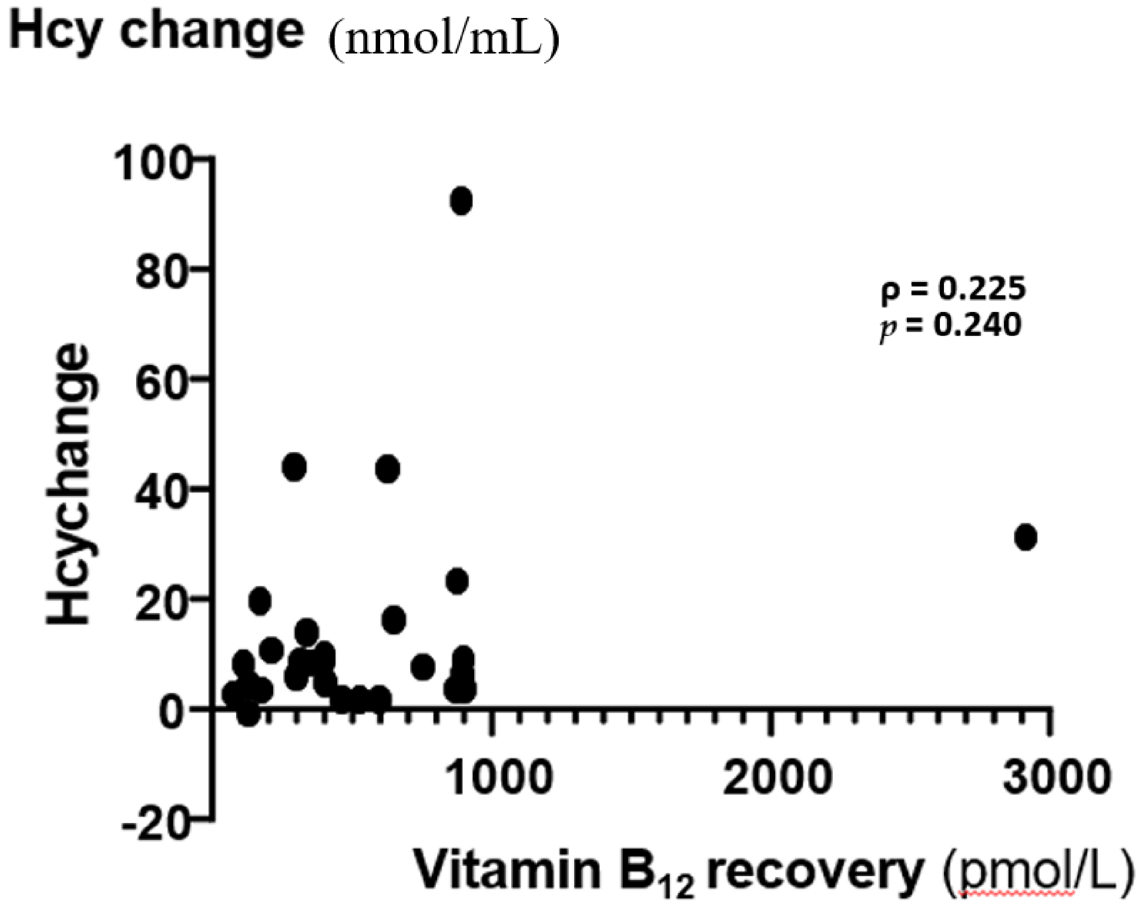

Supplementary Figure S3. There was no correlation between the degree of homocysteine (Hcy) decrease and change of vitamin B_{12} (ρ= 0.225, p = 0.240) was analyzed by the Spearman's rank correlation coefficient because the data of Hcy recovery and vitamin B_{12} recovery deviated from a normal distribution.

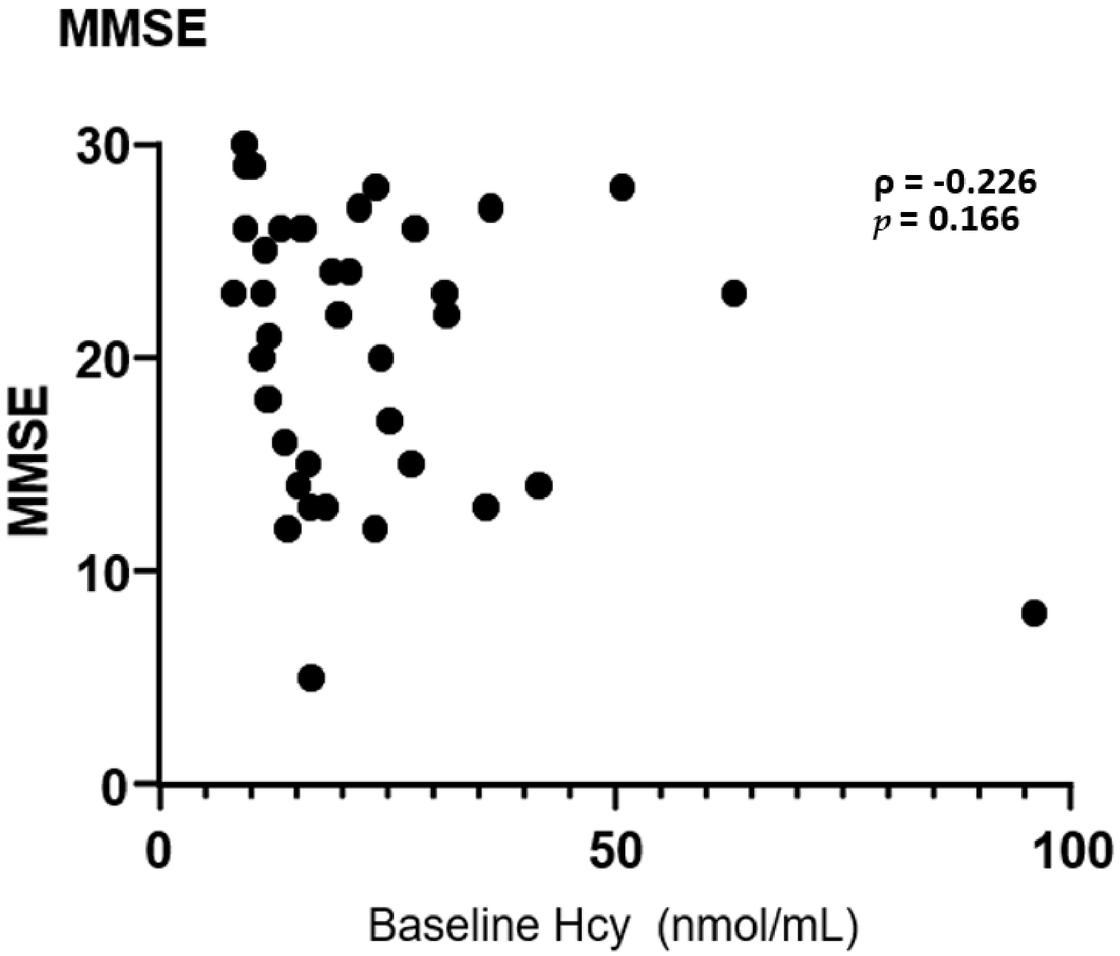

Supplementary Figure S4. No correlation is observed between baseline Mini-Mental State Examination (MMSE) score and baseline homocysteine (Hcy). $\rho = -0.226$, $p = 0.166$. Spearman's rank correlation coefficient (ρ) was used because the data of Hcy deviated from a normal distribution

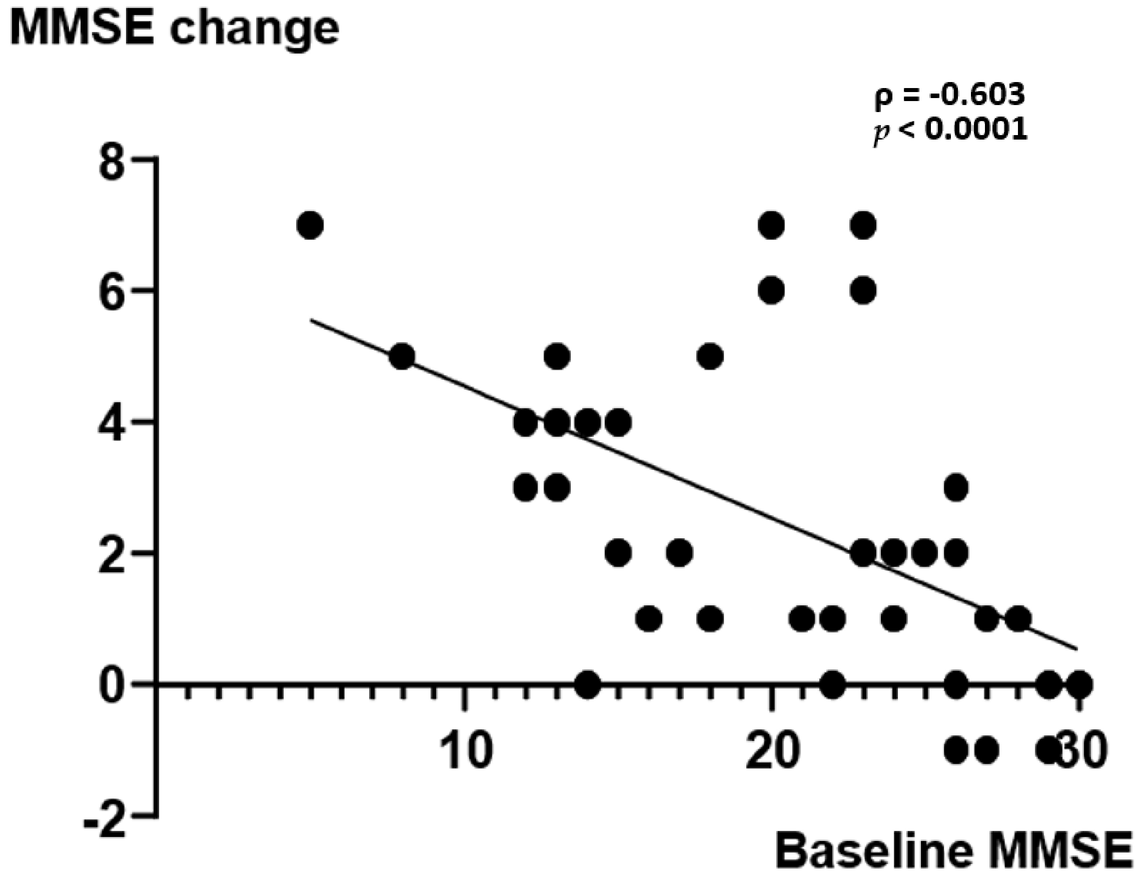

Supplementary Figure S5. Significant negative correlation is shown between baseline Mini-Mental State Examination (MMSE) score and degree of MMSE change following vitamin B_{12} supplementation (ϱ= -0.603, $p < 0.0001$). Spearman's rank correlation coefficient (ϱ) was used because the data of MMSE deviated from a normal distribution.

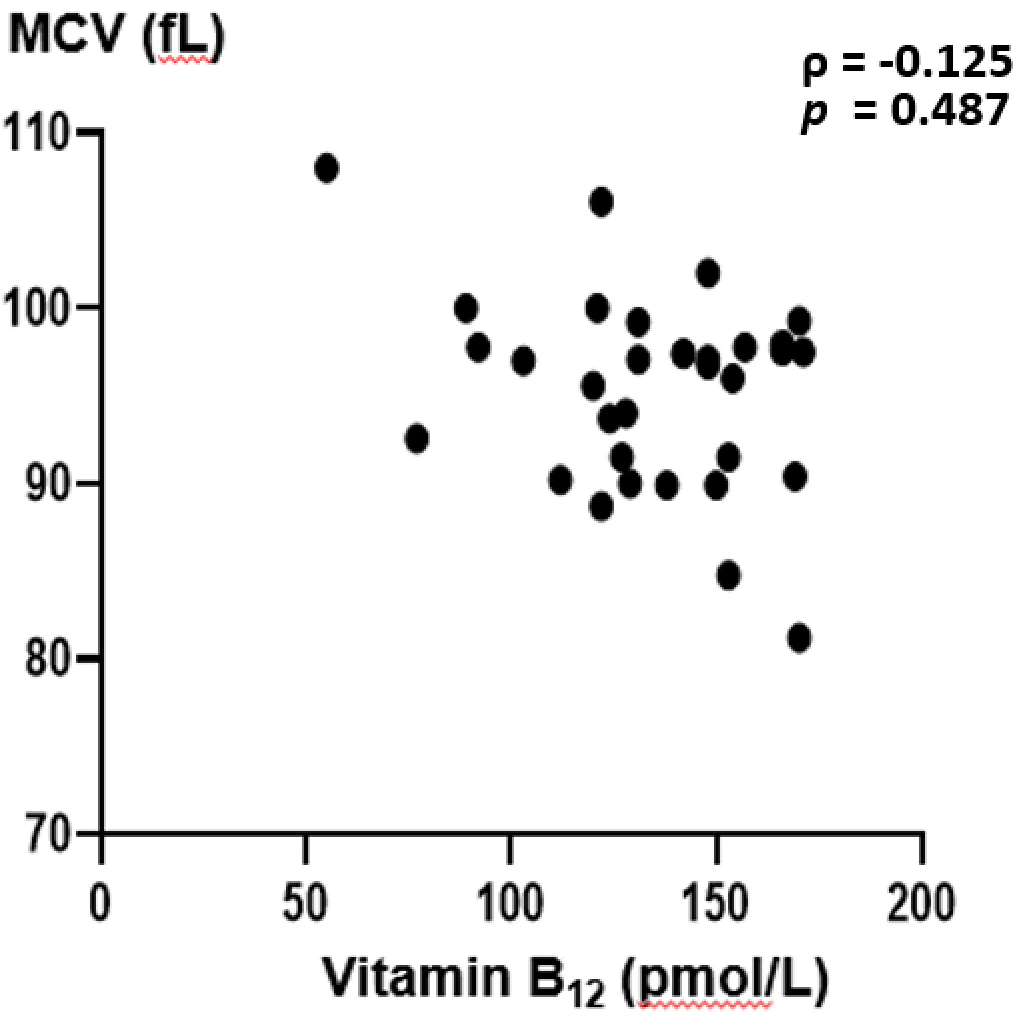

Supplementary Figure S6. There is no significant correlation between vitamin B_{12} concentration and mean corpuscular volume (MCV) (ϱ = -0.125, *p* = 0.487). Spearman's rank correlation coefficient (*ρ*) was used because the data of vitamin B_{12} deviated from a normal distribution

Suppplementary Table S1 Comparison of patient demographic data

	**Included (*n* = 39)**	**Not included (*n* = 44)**	*p*
Age (Mean ± SD)	80.1±8.2	79.9±11.3	0.739
Male sex, n	22 (56%)	27 (61%)	0.649
MMSE (Median(IQR）)	22 (15-26)	21(16-24)	0.531
Vitamin B_{12}(Median(IQR）), pmol/L	142 (122-154)	143 (124-157)	0.715
Folate (Median(IQR）), pg/mL	6.8 (5.0-8.4)	4.8 (3.1-8.2)	0.004
Hcy (Median(IQR）), nmoL/mL	16.7 (12.0-27.7)	19.2 (15.1-28.9)	0.348
MCV (Median(IQR）), fL	97.0 (91.0-97.9)	97.9 (93.7-100.0)	0.310
VSRAD z-score (Median(IQR）)	1.7 (1.2-2.6)	2.5 (1.5-4.2)	0.029
Education (Year) (Median (IQR）)	12 (8-12)	9 (7.8-12)	0.362

Comparison of demographic data between patients included in this study (*n* = 39) and those not included (*n* = 44). Abbreviations: Hcy, homocysteine; MMSE, Mini-Mental State Examination; (IQR); IQR, interquartile range; VSRAD, voxel-based specific regional analysis system developed for the study of Alzheimer’s disease

Suppplementary Table S2 The detailed data of 39 patients

No.	Age/ Sex	Education (Years)	VitB12 (pmol/L)		Folate (ng/mL)	Hcy (nmol/mL)		MCV (fL)	MMSE					Interval (Days)	Follow up Periods (Months)	MRI
			Pre	Post	Pre	Pre	Post	Pre	Pre	Post	0.5 (years)	1 (years)	2 (years)			VSRAD
1	75/M	12	153	>1107	53.0	23.8	15.0	91.5	28	29	NA	NA	NA	112	10.7	1.60
2	66/M	16	92	753	17.5	50.8	7.2	97.8	28	29	29	29	29	56	>24	NE
3	76/M	8	55	367	13.3	63.0	18.9	108.0	23	29	27	28	NA	28	>24	2.91
4	79/F	12	124	567	11.6	15.6	6.9	93.7	26	26	26	26	23	56	>24	NE
5	88/M	12	120	NE	10.5	16.5	9.9	95.6	13	17	16	15	13	56	>24	2.06
6	85/M	12	121	567	11.6	11.3	6.7	100.0	20	26	24	16	18	30	>24	1.71
7	84/F	12	141	NE	10.0	11.7	NE	NE	25	27	NA	NA	NA	77	>24	1.23
8	79/F	NE	89	1012	9.7	96.0	3.7	100.0	8	13	NA	NA	NA	84	8	NE
9	75/M	9	138	266	9.3	10.2	7.7	89.9	29	29	29	30	28	69	>24	1.96
10	87/M	NE	157	>1107	8.4	13.8	7.4	97.8	16	17	18	18	NA	49	20	1.69
11	75/F	12	146	NE	8.4	18.9	NE	NE	24	26	NA	NA	NA	35	1	NE
12	87/M	8	104	3054	8.3	41.7	10.4	NE	14	18	NA	NA	NA	84	>24	3.59
13	68/M	6	148	NE	8.1	13.3	NE	97.1	26	28	NA	NA	NA	79	15	NE
14	88/F	NE	170	>1107	7.5	15.3	11.7	81.2	14	14	14	12	NA	56	>24	3.09
15	71/M	12	142	NE	7.4	9.5	NE	97.4	29	28	28	28	27	56	>24	NE
16	84/F	9	112	323	6.8	35.8	16.2	90.2	13	16	14	11	NA	28	>24	2.51
17	81/F	8	167	NE	6.7	8.2	NE	NE	23	25	NA	NA	NA	84	>24	NE
18	77/M	8	166	NE	6.6	11.8	NE	97.6	18	19	NA	NA	NA	35	2	NE
19	87/M	8	166	434	6.4	24.3	13.8	97.9	20	27	NA	NA	NA	56	5	1.04
20	90/F	NE	142	NE	6.4	16.3	NE	NE	15	17	NA	NA	NA	84	4	NE
21	79/M	16	167	NE	6.4	11.4	9.5	NE	23	25	23	21	20	35	>24	2.09
22	89/F	12	131	496	6.1	21.9	13.4	97.1	27	26	25	24	23	133	>24	1.27
23	84/M	8	148	>1107	5.3	12.0	8.4	102.0	21	22	21	18	17	70	>24	NE
24	75/F	12	150	379	5.3	9.4	6.0	89.9	30	30	30	30	30	56	>24	1.19
25	91/F	8	128	300	5.0	14.1	15.0	94.0	12	16	14	14	15	49	>24	NE
26	80/M	9	154	510	4.9	20.8	15.1	96.0	24	25	NA	NA	NA	52	>24	1.10
27	78/M	NE	153	283	4.9	27.7	NE	84.8	15	19	NA	NA	NA	28	9	NE
28	87/F	NE	103	639	4.8	31.5	NE	97.0	22	22	NA	NA	NA	84	8	NE
29	90/F	8	148	602	4.7	23.7	14.2	96.8	12	15	NA	15	NA	21	18	2.31
30	78/M	8	122	760	4.4	12.0	10.4	106.1	18	23	22	20	19	86	>24	NE
31	84/M	NE	131	930	4.2	19.7	12.1	99.2	22	23	21	17	13	56	>24	1.03
32	49/M	12	77	753	3.8	28.1	11.8	92.6	26	28	NA	NA	NA	120	>24	1.23
33	75/F	12	169	>1107	6.3	36.3	13.1	90.4	27	28	27	27	27	77	>24	0.77
34	77/F	12	170	364	7.3	15.9	11.6	99.3	26	29	30	NA	NA	43	11	3.18

35	81/F	12	129	640	7.6	16.7	15.0	90.0	5	12	NA	NA	NA	77	10	1.55
36	89/M	NE	122	278	4.9	25.3	17.3	88.7	17	19	NA	NA	NA	28	4	2.62
37	85/F	NE	171	586	6.9	18.3	10.0	97.5	13	18	NA	NA	NA	NA	>24	3.92
38	79/M	9	127	511	4.4	31.3	17.4	91.5	23	30	29	28	27	67	>24	NE
39	71/M	12	142	720	7.4	9.5	7.8	97.4	26	25	25	25	25	56	>24	NE

Data of all 39 patients; age, sex, laboratory tests for vitamin B12, folate, Hcy, and MMSE score. MCV was performed for 33 and MRI VSRAD was performed for 23 patients. Abbreviations: Hcy, homocysteine, MCV, mean corpuscular volume; MMSE, Mini-Mental State Examination; MRI, magnetic resonance imaging; VSRAD, voxel based specific regional analysis system developed for the study of Alzheimer's disease; NE, not examined; NA, not available; M, male; F, female.
